# Analysis of Endophyte Diversity of *Rheum palmatum* from Different Production Areas in Gansu Province of China and the Association with Secondary Metabolite

**DOI:** 10.3390/microorganisms9050978

**Published:** 2021-04-30

**Authors:** Dawei Chen, Lingyun Jia, Qinzheng Hou, Xiang Zhao, Kun Sun

**Affiliations:** College of Life Sciences, Northwest Normal University, Lanzhou 730070, China; gansudaweichen@126.com (D.C.); lingyunjia1982@126.com (L.J.); hou_qzh@163.com (Q.H.); zhaoxiang199411@163.com (X.Z.)

**Keywords:** *R. palmatum*, endophytes, diversity, correlation analysis, ecological function

## Abstract

Investigations of the differences in the metabolites of medicinal plants have typically focused on the effects of external environmental factors. However, little is known about the relationship between endophytes diversity and host metabolites. We used high-throughput sequencing methods to compare the endophyte diversity of *Rheum palmatum* from eight different production areas in Gansu Province of China and to analyze the association between those areas and five secondary metabolites (aloe-emodin, rhein, emodin, chrysophanol, and physcion). The results show that the diversity and OTUs (Operational taxonomic units) abundance of endophytic fungi and bacteria of *R. palmatum* differed according to production area. Spearman analysis showed that the five secondary metabolites of *R. palmatum* were positively correlated with the diversity and abundance of endophytic fungi. Comparing both space and environmental differences to determine influences on community structure, VPA analysis revealed that geographic factors explained more difference in community composition of fungal and bacterial endophytes than climate factors. PICRUSt and FUNGuild predictive analysis indicated that metabolites were the primary components of endophytic bacteria in all samples, while the function of endophytic fungi was composed of dominant trophic modes (saprotroph and pathotroph), and relative abundances were different. Our results help elucidate the correlation of plant–microbe interactions and offer pivotal information to reveal the role of endophytes in the production of *R. palmatum* and its important secondary metabolite.

## 1. Introduction

Endophytes are a phenomenon of the symbiosis between plants and the microorganisms that inhabit the internal tissues of plants, but they do not cause any symptoms of disease for plant. Endophytes always play an important role in plant growth, development, abiotic and biotic stress tolerance, and adaptation in nature. Our understanding of endophytes and their ecological aspects are overtly limited, and we have only recently started to appreciate their essence [1]. Endophytes can also be used for improving the production of agricultural and commercial plants [2]. Park et al. [3] reported that plant disease caused by *Botrytis cinerea* and *Cylindrocarpon destructans* can be significantly reduced in *Panax ginseng* by inoculating endophytic *Trichoderma citrinoviride*. Marcia et al. [4] found that inoculation of endophytic *Penicillium* can improve photosystem II efficiency, leaf nitrogen, and carbohydrate content, promoting growth of *Prosopis chilensis*. Meanwhile, endophytes can regulate secondary metabolite accumulation of medicinal plants; they are not only used as reservoirs of new bioactive secondary metabolites but also as a potential substitute for the secondary metabolites of medicinal plants [5]. Song et al. [6] reported that endophytic *Bacillus altitudinis* enhanced ginsenoside accumulation, which was isolated from *Panax ginseng*. Stierle et al. [7] first reported that endophytic fungi isolated from *Taxus* sp. could produce paclitaxel, a medicinal ingredient with an anticancer effect. Thus, endophytes play an important role in growth-promotion, accumulation of metabolites, and the search for substitutes of medicinal plants [8].

*Rheum palmatum*, a well-known traditional Chinese medicine [9], has been used for medical purposes by the Chinese for thousands of years [9]. The *Divine Farmer’s Herb-Root Classic* described that it has been used for the treatment of many diseases, such as constipation, jaundice, ulcers, and gastrointestinal hemorrhage [10]. The roots are rich in anthraquinones, whose major components are aloe-emodin, rhein, emodin, chrysophanol, and physcion, which have been extensively investigated because of their excellent bioactivity. Anthraquinones have anti-inflammatory, antitumor, antiviral, anti-obesity, and anticoagulant properties [11]. In recent years, the wild stock of this species has decreased more rapidly than ever, and most natural populations have been destroyed in order to meet the commercial demand for the rhubarb species [12]. Efforts are being made to understand the biology of the crop and to replace the traditional approaches of *R. palmatum* cultivation with scientific practices.

Many reports have shown that the secondary metabolites of medicinal plants from different areas are different [13]. At present, much of the research has focused on the impact of the external ecological environment on medicinal plants, such as temperature, rainfall, light, and so on [14]. However, the effect of the internal environment of medicinal plants has seldomly been studied. In particular, endophytes compose part of the microecosystem in the internal environment of medicinal plants and may help explain from a new perspective the differences in quality between *R. palmatum* from different producing areas.

Gansu province is main production area of *R. palmatum* [15]. In this study, the primary goals of this paper were as follows: (1) compare endophytes diversity of *R. palmatum* from different areas in main production area and explore the external environment differences to determine influences on community structure; (2) analyze the relationship between endophytes and host metabolites; and (3) predict the endophytic bacterial and fungal functions of *R. palmatum*. These results may lay a foundation for expanding the knowledge of plant–microbe relationships and may contribute to a boost in *R. palmatum* quality.

## 2. Materials and Methods

### 2.1. Experimental Materials

**Figure 1:**
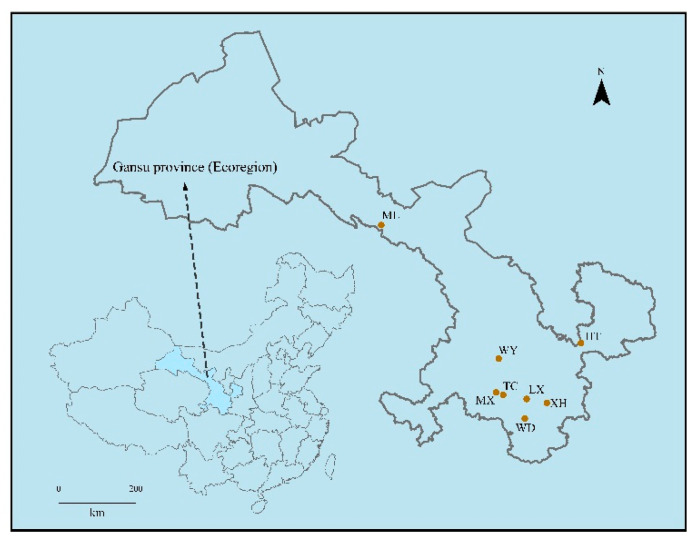
Simplified map of sampling area showing location.

The 2-year-old roots of *R. palmatum* were collected from eight different production areas in Gansu Province, China (Table 1 and Figure 1). All samples were separated and washed with running tap water, then rinsed three times with distilled water. A single sample consisted of 0.5 g root as one sample. The samples were successively immersed in 75% ethanol for 5 min, 2.5% NaClO solution for 1–2 min, and 75% ethanol for 1 min, and then were rinsed five times in sterile water. The last portion of the washing water was inoculated in PDA (potato dextrose agar) at 28 °C for 10 d and NA (nutrient agar) at 37 °C for 3 d to validate sterilization efficiency. All samples were stored at −80 °C until DNA extraction [16].

### 2.2. DNA Extraction, Polymerase Chain Reaction (PCR) Amplifcation, and Sequence Processing

The total genomic DNA was extracted from all samples by using the MOBIO Power -Soil^®^ Kit (MOBIO Laboratories, Inc., Carlsbad, CA, USA), according to the manufacturer’s instructions. The DNA extracts were analyzed for their concentration using NanoDrop spectrophotometer (Termo Fisher Scientifc, Model 2000, Waltham, MA, USA) and stored at −20 °C for further PCR amplification. The PCR assays were performed in 20 μL mixture containing 4 μL of 5× Fast-Pfu buffer, 2 μL of 2.5 mM dNTPs, 0.8 μL of each primer (5 μM), 0.4 μL of FastPfu Polymerase, 10 ng of template DNA, and ddH_2_O. Bacterial 16S gene was amplified with primers 338F (5′-ACTCCTACGGGAGGCAGCA-3′) and 806R (5′-GGACTACHVGGGTWTCTAAT-3′). Amplification was performed under the following conditions: initial denaturation at 95 °C for 3 min, 30 cycles at 95 °C for 30 s, 52 °C for 30 s, and 72 °C for 45 s and a final extension at 72 °C for 5 min. The fungal ITS genes were amplified using the primers ITS1F (5′-CTTGGTCATTTAGAGGAAGTA -A-3′) and ITS2R (5′-GCTGCGTTCTTCATCGATGC-3′). The PCR reactions were conducted using the following program: 3 min of denaturation at 95 °C, 35 cycles of 30 s at 95 °C, 30 s for annealing at 55 °C, and 45 s for elongation at 72 °C and a final extension at 72 °C for 10 min. The PCR products were analyzed by agarose gel electrophoresis. For each sample, three successful PCR products were pooled and purified using EasyPureTM PCR Clean up/Gel Extraction Kit (Axygen Biosciences, Union City, CA, USA) according to manufacturer’s instructions. Purified amplicons were sequenced on an Illumina NovaSeq platform for paired-ends according to the standard protocols [17].

### 2.3. Metabolites of R. palmatum Quantitative Analysis

Standard aloe-emodi, rhein, emodin, chrysophanol, and physcion were obtained from Shanghai R&D Center for Standardization of Traditional Chinese Medicines. High-performance liquid chromatography (HPLC)-ultrapure water, analytical-grade methanol, NaHCO_3_, and phosphoric acid were purchased from Sangon Biotech, Ltd. (Shanghai, China).

According to the *Chinese Pharmacopoeia* (Edition 2015) [18], the dried root of each treatment specimen (three replications) was pulverized and sieved through a 300 μm mesh. A total of 1.5 g of powder of each sample was precisely weighed, added to 10 mL 0.1% NaHCO_3_ aqueous solution, and treated with ultrasound (30~40 °C, 700 W) for 20 min. Then, 40 mL methanol was added for ultrasonic treatment for 50 min. Filtrate was obtained by filtration of 0.22 μm Millipore filter unit, and 10 μL of sample solution was injected into HPLC for determination.

According to method of Chen et al. [19], the samples were analyzed by HPLC (Waters) using C18 (4.6 × 250 mm, 5.0 μm, Waters E2695, Milford, MA, USA) at 30 °C, and the content of metabolites was determined: The mobile phase was methanol −0.1% phosphoric acid (80:20). The flow rate was 1 mL·min^−1^. The detection wavelength was 254 nm.

### 2.4. Data Analysis

The data were analyzed by utilizing the QIIME pipeline, as previously performed in [20]. Fungal and bacterial sequences were trimmed and assigned to each sample based on their barcodes. The UPARSE-OTUref was used to classify OTUs at the species level by searching all sequences against the Silva bacterial 16S database [21]. OTUs were classified at the species level by searching against the UNITE fungal database [22]. Sequences were binned into operational taxonomic units (OTUs) at 97% similarity level by using USEARCH software (http://drive 5.com/uparse/, accessed on 14 November 2020) [21]. Rarefaction analysis based on Mothur v.1.21.1 was conducted to reveal the diversity indices, including goods coverage, Chao 1, and Shannon [23]. Unweighted pair group method with arithmetic mean (UPGMA) cluster analysis was used to analyze the community differences between different samples based on weighted unifrac distances [24]. Correlation analysis between metabolites and diversity of endophytes used the Spearman method [23]. The contributions of climate (mean annual precipitation (MAP), mean annual temperature (MAT)), space (longitude, latitude, and altitude), and their interactions to the variation in the fungal and bacterial endophytic community were calculated by variance partitioning analysis (VPA) [25]. Metabolic and ecologically relevant functions were annotated by PICRUSt for the 16S rDNA OTU and FUNGuild v1.0 for the ITS OTU [26].

## 3. Results

### 3.1. Surface-Sterilization Efficiency

After a certain period of cultivation, no colonies were observed in PDA and NA medium, which indicated that surface-sterilization was effective and could be used in subsequent experiments.

### 3.2. Analysis of Sequencing Data and Alpha Diversity

**Figure 2:**
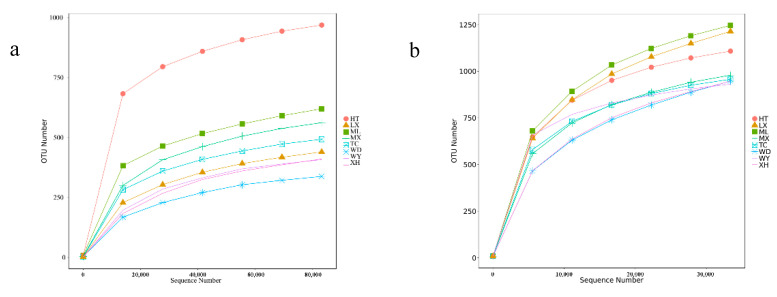
Rarefaction curves base on pyrosequencing of endophytic fungi (**a**) and bacteria (**b**) for each sample.

The rarefaction curve can reflect the variation of species diversity and the richness of samples with the sequencing amount [26,27]. With the increase in the amount of sequencing effort, the rarefaction curves of the samples based on the number of species observed became stable, which indicated that the amount of sequencing data was gradually becoming reasonable (Figure 2).

A total of 707,382 and 605,179 effective tags were obtained for the fungal and bacterial samples, respectively, after filtering and removing chimeric particles, with the library coverage of the samples being higher than 0.988, which indicates that the sequencing data confidently reflected the structure of the endophytic fungi and bacteria community of the samples. Alpha diversity indices (Chao1 and Shannon’s diversity index) presented differences among all samples of *R. palmatum*. Chao1 indicated a fungal and bacterial community richness trend of HT > ML > MX > TC > LX > XH > WY > WD and WD > ML > LX > XH > MX > TC > HT > WY, respectively. Shannon’s diversity index revealed a fungal and bacterial community diversity trend of HT > ML > MX > TC > XH > WD > LX > WY and WY > ML > MX > LX > TC > HT > WD > XH, respectively (Table 2).

### 3.3. Community Composition

**Figure 3:**
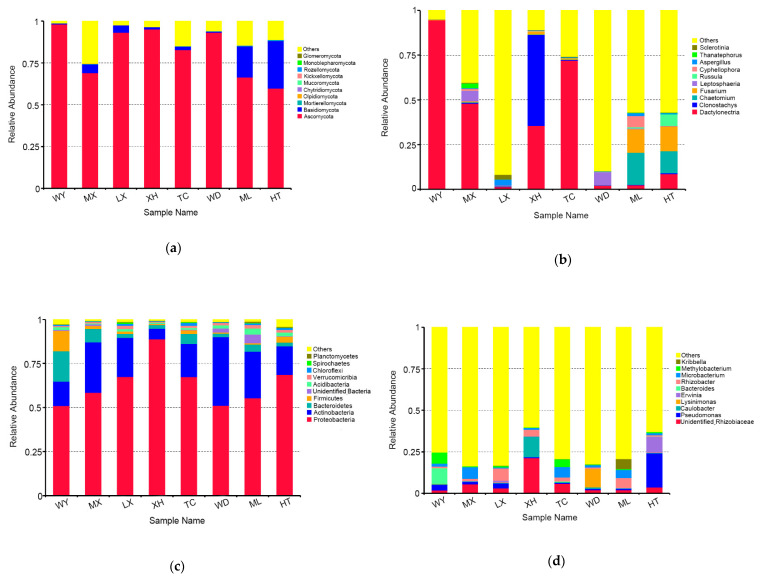
Relative abundances of the endophytic fungi at the phylum level (**a**), endophytic fungi at the genus level (**b**), endophytic bacteria at the phylum level (**c**), endophytic bacteria at the genus level (**d**) for each sample. Relative abundances are based on the proportional frequencies of the DNA sequences that could be classified. “Other” represents the total of relative abundance outside the top ten maximum relative abundance levels.

The fungal OTUs were assigned to 11 phyla and 265 genera. The dominant fungal phylum across all of samples was Ascomycota, with relative abundances ranging from 59.85 % to 98.09%. At the genus level, *Dactylonectria* was the dominant genus in three samples (WY, MX, TC), with relative abundances ranging from 47.86% to 94.62%. *Clonostachys* was the dominant genus in the XH sample (50.90%). *Leptosphaeria* was the dominant genus in the WD sample (7.43%). *Chaetomium* was the dominant genus in the ML sample (17.97%). *Fusarium* was the dominant genus in the HT sample (13.91%). *Aspergillus* was the dominant genus in the LX sample (3.24%) (Figure 3a,b).

The bacterial OTUs were assigned into 25 phyla and 472 genera. The dominant bacterial phylum across all of samples was Proteobacteria, with relative abundances ranging from 51.00 % to 88.87%. At the genus level, *Bacteroides* was the dominant genus in the sample of WY (9.50%), *Microbacterium* was the dominant genus in the sample of MX and TC (6.91%, 6.35%, respectively), *Rhizobacter* was the dominant genus in the sample of LX and ML (7.36%, 5.97%, respectively), unidentified *Rhizobiaceae* was the dominant genus in the sample of XH (21.24%), *Lysinimonas* was the dominant genus in the sample of WD (11.21%), and *Pseudomonas* was the dominant genus in the sample of HT (20.61%) (Figure 3c,d).

**Figure 4:**
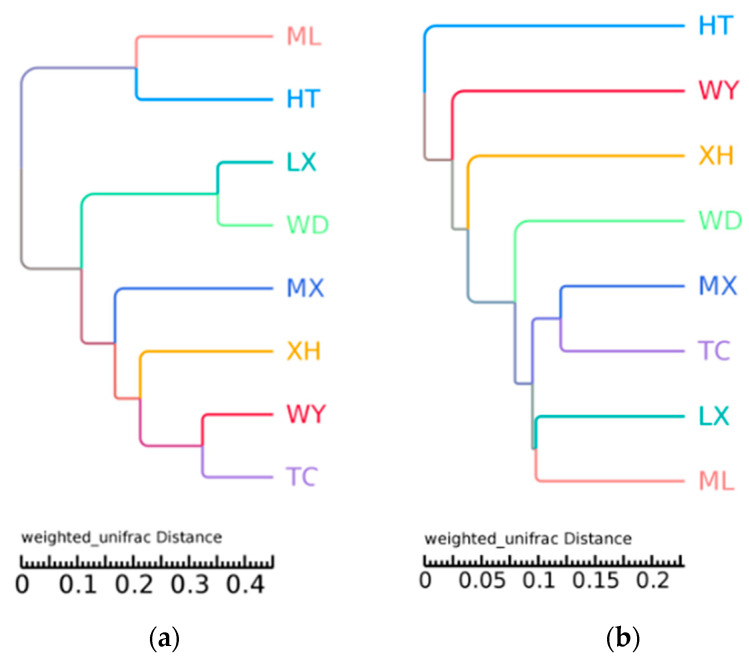
UPGMA tree of different fungal (**a**) and bacterial (**b**) community structures at the phylum level in the different samples.

The UPGMA clustering results of fungal communities showed that ML and HT, LX and WD, WY and TC samples clustered together, exhibiting a relatively similar community structure (Figure 4a). The results of bacterial communities showed that MX and TC samples clustered together, as did LX and ML, exhibiting a relatively similar community structure (Figure 4b).

### 3.4. Effects of Climate and Space on Fungal Endophytic Community

**Figure 5:**
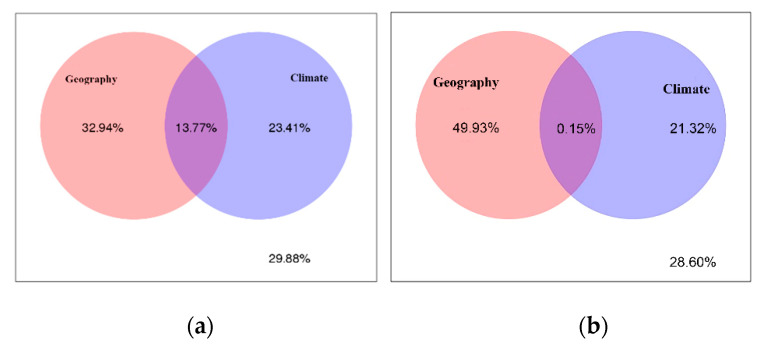
Variance partitioning analysis (VPA) of geographical versus climate factor on endophytic fungal (**a**) and endophytic bacterial (**b**) community composition.

We found that geography and climate explained unique variation in *R. palmatum* fungal (32.94% and 23.41%) and bacterial (49.93% and 21.32%) endophytic community composition, respectively. The overlap of the two sets of predictors explained 13.77% and 0.15% of the variation of fungi and bacteria, respectively. The rest of the unexplained parts of fungi and bacteria were 29.88 % and 28.60%, respectively (Figure 5).

### 3.5. Correlation Analysis between Endophytes Diversity and Metabolites of R. palmatum

**Figure 6:**
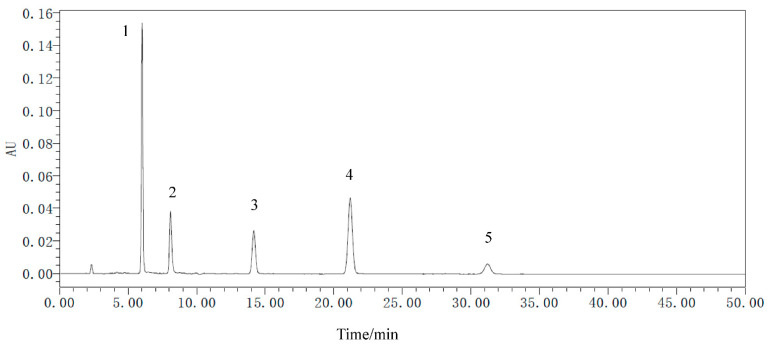
The HPLC of metabolite standards of *R. palmatum*. 1 is aloe-emodin, 2 is rhein, 3 is emodin, 4 is chrysophanol, 5 is physcion.

**Figure 7:**
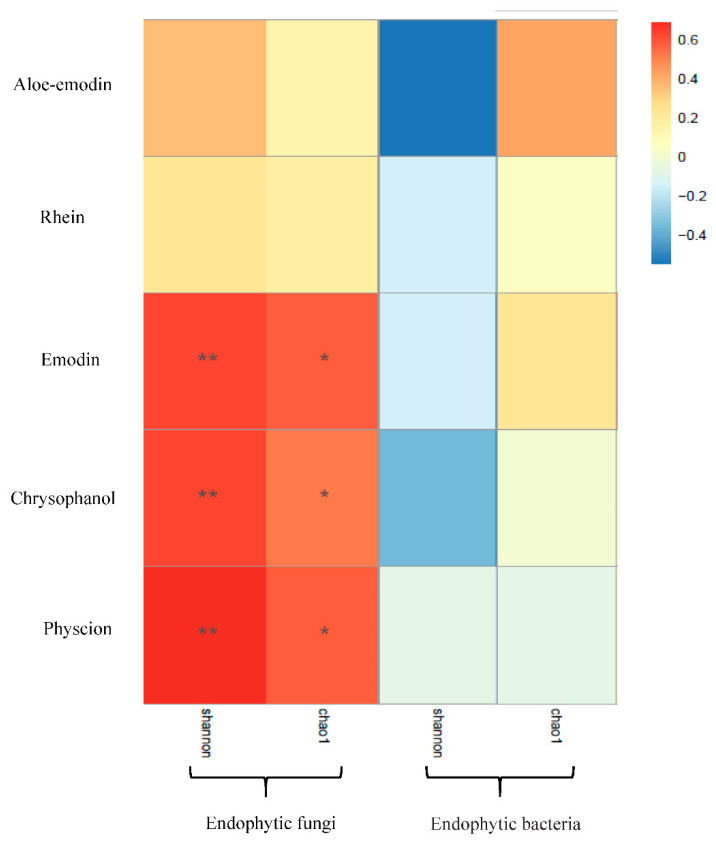
Correlation analysis between metabolites and diversity of endophytes. **Note:** * indicate the differences are significant at *p* < 0.05 and ** indicate the differences are significant at *p* < 0.01.

Five secondary metabolites standards of *R. palmatum* by HPLC as shown in Figure 6, it indicated that five secondary metabolites of *R. palmatum* can be effectively tested under the condition of this HPLC. Metabolites of *R. palmatum* differed according to production area (Table 3). Correlation analysis showed that the overall contents of five metabolites were positively correlated with Shannon’s diversity index and Chao1 of endophytic fungi, among which, the specific content of emodin, chrysophanol, and physcion was significantly positively correlated with endophytic fungal Shannon’s diversity index and Chao1. While the contents of five metabolites were negatively correlated with Shannon’s diversity index of endophytic bacteria, the content of aloe-emodi, rhein, and emodin were positively correlated with the Chao1 of endophytic bacteria, while the content of chrysophanol was not correlated with Chao1 of endophytic bacteria and the content of physcion was negatively correlated with Chao1 of endophytic bacteria (Figure 7).

### 3.6. PICRUSt and FUNGuild Functional Prediction Analysis

**Figure 8:**
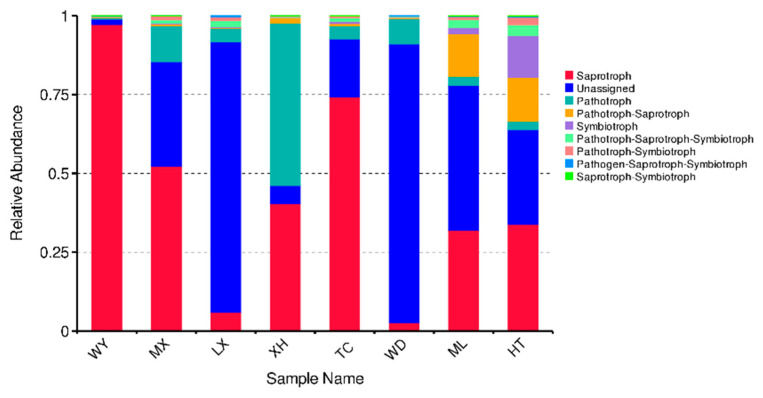
Relative abundance of predicted trophic mode of fungi.

FUNGuild was used to predict the nutritional and functional groups of the fungal communities with different samples. The results show that eight trophic mode groups could be classified, including pathogen-saprotroph-symbiotroph, pathotroph, pathotroph-saprotroph, pathotroph-saprotroph-symbiotroph, pathotroph-symbiotroph, saprotroph, saprotroph-symbiotroph, symbiotroph. While all of the OTUs that were not matching with any taxa in the database were categorized as “unassigned”. Saprotroph was dominant trophic mode in six samples (WY, MX, LX, TC, ML, HT), with relative abundances ranging from 5.91% to 97.24%, while pathotroph was the dominant trophic mode in XH and WD sample (51.53% and 8.07%, respectively) (Figure 8).

**Figure 9:**
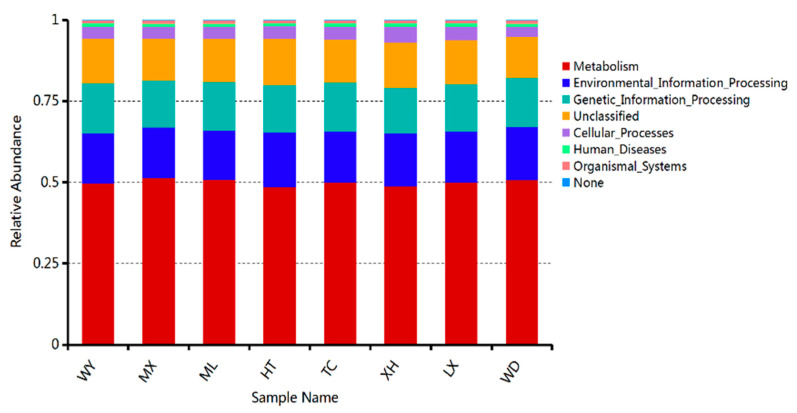
Relative abundance of predicted KEGG Orthologs functional profiles (KEGG level 1) of bacteria.

To study bacterial function, we used PICRUSt to perform bacterial function prediction analysis. Through comparisons with the Kyoto Encyclopedia of Genes and Genomes (KEGG) database, six categories of biological metabolic pathways (primary functional level) were obtained, which included metabolism, genetic information processing, environmental information processing, cellular processes, organ systems, and human diseases. The metabolism pathway was the primary component in all samples, accounting for 48.65–51.37% (Figure 9).

## 4. Discussion

In this study, the dominant fungal and bacterial phyla across all of samples were Ascomycota and Proteobacteria, respectively. These two phyla were distributed in each sample, but their relative richness was different. Many studies have shown that Ascomycota and Proteobacteria were, respectively, the dominant phyla of fungal and bacterial endophytes in many plants [28,29,30]. At the genus level, dominant genera of endophytes and their relative richness was different, which may be caused by differences in altitude, mean annual precipitation, and mean annual temperature of different production areas, which may lead to differences in endophytic bacteria and fungi at the genus level [31]. To explore the effects of external environment on the differences of endophytic fungi and bacterial communities in *R. palmatum,* the contributions of climate, geography, and their interactions on the variation in the fungal and bacterial endophytic community were calculated by VPA analysis, which showed that spatial distance played a stronger role in structuring endophytic bacterial and fungal communities than climate. U’Ren et al. [31] reported that climate was a better predictor than spatial distance for foliar endophyte communities at a continental scale. Further, they found that endophyte isolation frequency increased as a function of both growing season length and annual precipitation, suggesting that climate plays a strong role in structuring these communities. The reason for this conclusion may be due to the different plant species concerned or the different methods used to study the diversity of endophyte. U’Ren et al. [31] used culture-based methods in their study, which do not capture as much diversity as culture-free methods, and thus may have failed to detect certain taxa.

Endophytes have a wide range of biosynthesis ability and can produce a variety of secondary metabolites with biological activities. Many studies have reported that endophytes can produce the same or similar substances as host secondary metabolites [7,32,33]. This study investigated the relationships between endophytic fungal and bacterial diversity and host secondary metabolites. Spearman correlation analysis showed that secondary metabolites of *R. palmatum* were positively correlated with endophytic fungal diversity and abundance, which is consistent with the results of Cui et al. [34], who reported significant correlations between metabolites of *Cynomorium songaricum* and endophytic fungi, but the relationship between endophytic bacteria and host was ignored in their study. This preliminary conclusion suggests that endophytic fungi of *R. palmatum* were used as reservoirs of new bioactive secondary metabolites and also as potential substitutes for secondary metabolites of *R. palmatum.*

PICRUSt analysis can predict the metabolic function of bacterial communities with high reliability [35], and it has been used to study bacterial functions in many plants [36]. We performed PICRUSt functional predictive analysis using the high-throughput sequencing results. The results show that the endophytic bacteria of *R.palmatum* primarily comprised six functional layers in all samples. Among these functional layers, metabolism was the primary component in all samples. This result is similar to that obtained in a study of the function of barley and tomato rhizosphere bacteria by Pii et al. [37]. Pepe-Ranney et al. [38] reported that endophytes are derived from the rhizosphere microbiome, leading to results that are similar. Because of the limitations of PICRUSt functional prediction analysis, we cannot reveal how endophytes affect plant metabolism through functional prediction and so on. This study only preliminarily predicted the functions of related bacteria. In future studies, further validation should use methods such as metagenomics, which can help to better understand the endophytic bacteria function of *R. palmatum*.

FUNGuild is often used for comparisons of fungal functions and for identifying specific functional classifications of fungi. Recently, it was widely used in fungal community research [39]. In this study, we used FUNGuild to predict fungal endophytes of *R. palmatum*. The results show that the function of endophytic fungi was composed of dominant trophic modes (saprotroph and pathotroph), and relative abundances were different according to production area. This result is similar to that obtained in a study of Miscanthus rhizosphere and endorhizosphere functional groups studied by Martínez-Diz et al. [39]. Although FUNGuild has been used to analyze the function of fungi to some extent, the method has some limitations because it is based on existing literature and data. Therefore, in order to study comprehensively the function of endophytic fungi, we should further study the classification and functional groups of soil fungi.

## Figures and Tables

**Table 1:** The information of material and sample area.

Location	Altitude (m)	Longitude	Latitude	Mean Annual Precipitation (mm)	Mean Annual Temperature (°C)	Code No.
Weiyuan county, Dingxi city	2221	104°09′30.45″	35°04′11.43″	507	6.8	WY
Min county, Dingxi city	2489	104°02′08.94″	34°17′26.24″	635	5.5	MX
Li county, Wudu city	2282	104°52′38.03″	34°05′46.48″	488.2	9.9	LX
Xihe county, Wudu city	1849	105°26′02.39″	33°58′42.07″	533	8.4	XH
Tanchang county, Wudu city	2186	104°13′54.58″	34°13′21.42″	583.9	9.3	TC
Chiba township, Wudu city	2499	104°47′45.95″	33°38′37.19″	470	14.7	WD
Minle county, Zhangye city	2686	100°54′10.44″	38°16′44.33″	351	4.1	ML
Huating county, Pingliang city	1746	106°30′26.28″	35°18′22.18″	607	7.7	HT

Note: Mean annual precipitation and mean annual temperature were 30-year average climate variables.

**Table 2:** Community diversity of fungi and bacteria.

Sample	Endophytic Fungi				Endophytic Bacteria			
	Effective Tags	Shannon	Chao1	Goods_Coverage	Effective Tags	Shannon	Chao1	Goods_Coverage
WY	90,843	0.643	443.212	0.999	59,514	7.794	1107.833	0.995
MX	90,144	3.420	630.000	0.999	78,683	7.220	1414.852	0.992
LX	96,899	1.849	525.513	0.999	93,707	7.217	1732.563	0.989
XH	93,098	2.076	465.000	0.999	86,798	5.541	1580.968	0.990
TC	87,301	2.409	559.585	0.999	63,777	7.064	1291.762	0.993
WD	89,655	1.897	392.016	0.999	57,993	6.619	2120.333	0.988
ML	82,125	5.691	757.963	0.998	88,494	7.249	1913.009	0.988
HT	77,317	6.715	1057.724	0.998	76,213	6.871	1444.263	0.992

**Table 3:** Metabolite content of *R. palmatum* in different production areas.

Sample	Aloe-Emodin (mg/g)	Rhein (mg/g)	Emodin (mg/g)	Chrysophanol (mg/g)	Physcion (mg/g)
XH	0.61	1.23	1.88	5.24	1.28
LX	0.38	0.28	0.81	3.12	0.74
TC	1.38	2.33	0.94	4.16	1.58
MX	0.39	0.20	1.77	6.66	1.99
WY	0.36	0.40	0.22	2.31	0.97
WD	0.96	0.38	0.53	3.16	0.99
ML	0.62	2.02	3.47	4.96	1.84
HT	0.95	1.10	2.89	7.39	2.27

## Data Availability

The 16S rRNA and ITS gene sequences of endophytes used in this manuscript were submitted to the NCBI and the Accession number is SAMN18317781.
